# Usefulness of Surgical Media Center as a Cataract Surgery Educational Tool

**DOI:** 10.1155/2016/8435086

**Published:** 2016-01-10

**Authors:** Tomoichiro Ogawa, Takuya Shiba, Hiroshi Tsuneoka

**Affiliations:** Department of Ophthalmology, The Jikei University School of Medicine, 3-25-8 Nishishinbashi, Minato-ku, Tokyo 105-0003, Japan

## Abstract

*Purpose.* This study retrospectively analyzed cataract surgeries to examine the usefulness of Surgical Media Center (SMC) (Abbott Medical Optics Inc.), a new cataract surgery recording device, for training of cataract surgery.* Methods. *We studied five hundred cataract surgeries conducted with a phacoemulsification system connected to the SMC. After surgery, the surgical procedures were reviewed, with changes in aspiration rate, vacuum level, and phaco power displayed as graphs superimposed on the surgical video. We examined whether use of SMC is able to demonstrate the differences in technique between experienced and trainee operators, to identify inappropriate phacoemulsification techniques from analyzing the graphs, and to elucidate the cause of intraoperative complications.* Results*. Significant differences in the time taken to reach maximum vacuum and the speed of increase in vacuum during irrigation and aspiration were observed between experienced and trainee operators. Analysis of the graphs displayed by SMC detected inappropriate phacoemulsification techniques mostly in cases operated by trainee operators.* Conclusions*. Using SMC, it was possible to capture details of cataract surgery objectively. This recording device allows surgeons to review cataract surgery techniques and identify the cause of intraoperative complication and is a useful education tool for cataract surgery.

## 1. Introduction

**Figure 1:**
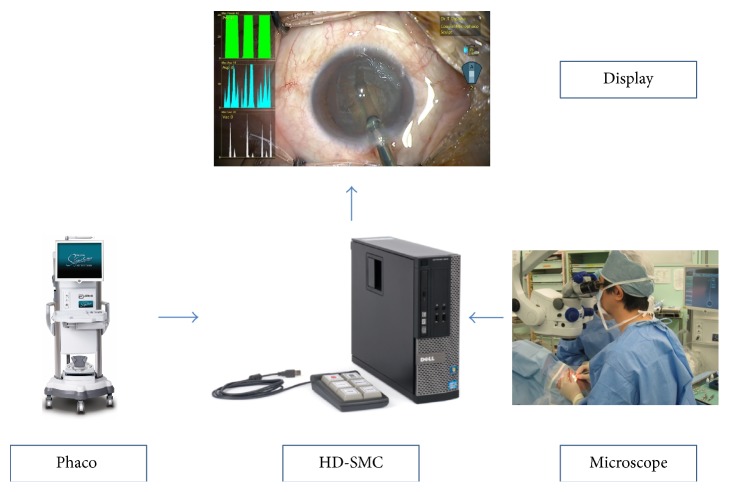
The High-Definition Surgical Media Center (HD-SMC) used with a phacoemulsification system.

**Figure 2:**
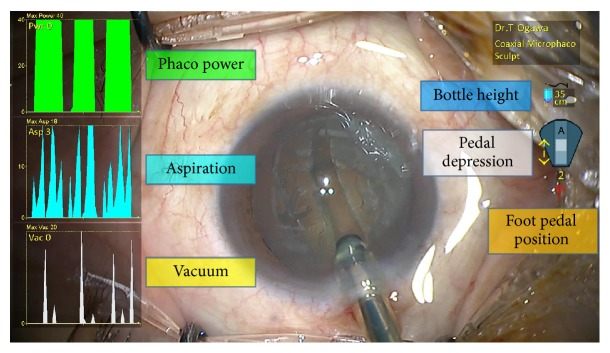
An example of display by the High-Definition Surgical Media Center (HD-SMC).

With technological advances in phacoemulsification instruments, emulsification and aspiration of the crystalline lens through even smaller incisions are now possible [1–6]. These instruments play very important roles in cataract surgeries. During surgery, information such as the amount of ultrasound generated by the instrument and the vacuum level achieved can be presented on the intraoperative video image using video overlay. However, the conventional overlay displays ultrasound power, vacuum pressure, and aspiration rate at one instant only, and it is difficult to capture how phaco power and vacuum change over time. To address this issue, a new generation cataract surgery recording device, the Surgical Media Center (SMC) (Abbott Medical Optics Inc.), was developed. The SMC is a cataract surgical data management tool designed for use with phaco systems such as Sovereign, Sovereign Compact, and Signature (Abbott Medical Optics Inc.). The SMC consists of a computer installed with dedicated software that records the intraoperative images from the surgical microscope together with phaco power, vacuum level, and aspiration rate over time during the surgery and displays the data as graphs synchronized with the surgical video (Figure 1). Since the graphs are recorded simultaneously with the surgical video, review of the video after surgery provides clear information of how the changes in phaco power, aspiration rate, and vacuum level over time affect the process of phacoemulsification. In addition, the state of foot pedal depression is also displayed, allowing surgeons to assess the surgical manipulations objectively (Figure 2). Recently, High-Definition Surgical Media Center (HD-SMC) (Abbott Medical Optics Inc.) with improved image quality has been launched, allowing detailed observation of cataract surgery procedures in high-definition images.

Surgical recording devices with the above-mentioned features should be widely used as an educational tool for learners of cataract surgery. However, there are few reports on detailed analysis of these devices and their educational usefulness in training cataract surgeons. In this study, we examined the practical advantages of the new cataract surgery recording devices, SMC and HD-SMC, and verified that these devices are useful educational tools for cataract surgery.

## 2. Patients and Methods

### 2.1. Patients

Three hundred and sixty-eight patients (500 eyes) who underwent cataract surgery using Sovereign, Sovereign Compact, or Signature at the Jikei Medical University School of Medicine between April 2008 and July 2014 were analyzed. Surgeries were conducted by three experienced cataract surgeons and three surgeons who were learning the surgical techniques (trainee operators).

### 2.2. Methods

Phacoemulsification was conducted using a phacoemulsification system (Sovereign, Sovereign Compact, or Signature) connected to a surgical microscope and a surgical data recording device (SMC or HD-SMC). After surgery, the changes in phaco power, vacuum level, and aspiration rate over time recorded by the SMC or HD-SMC were displayed in graphs synchronized with the surgical video.

An experienced operator was defined as a surgeon who had conducted cataract surgeries in over 300 eyes per year and in a total of over 2000 eyes. A trainee operator was defined as a surgeon who had conducted cataract surgeries in a total of less than 200 eyes. Six surgeons conducted cataract surgeries during the study period: three were experienced and three were trainees.

This study aimed to examine whether SMC or HD-SMC is able to demonstrate the difference in techniques between experienced and trainee operators, to identify inappropriate phacoemulsification techniques from analyzing the graphs, and to elucidate the cause of intraoperative complications such as posterior capsule rupture.

**Figure 3:**
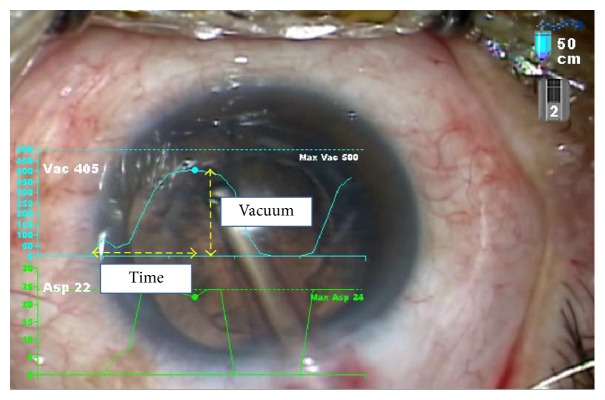
Changes in vacuum level over time during irrigation and aspiration (IA).

To compare the performance of experienced and trainee operators, 20 cases of cataract with grade 2 nucleus hardness (Emery-Little classification) performed by each of the 6 surgeons were selected. During irrigation and aspiration (IA), the time taken for vacuum to increase from 0 to a plateau and the peak vacuum level were measured three times. The speed of increase in vacuum was calculated by dividing maximum vacuum level by the time to each maximum vacuum (Figure 3). For Trainee Operator 1, maximum vacuum, time to maximum vacuum, and speed of increase in vacuum were determined for the subsequent 40 cataract surgeries. Statistical analysis was performed using Kruskal-Wallis test. A *p* value less than 0.05 was considered significant. The data were measured by a staff member not related to the operators or to the study.

In addition, the three experienced operators analyzed the relationship between the surgical video and surgical parameter data displayed in the overlays in cases, where phacoemulsification of nucleus or aspiration of cortex was difficult, and in cases with intraoperative complications.

## 3. Results

### 3.1. Comparison between Experienced and Trainee Operators

The speed of increase in vacuum level is shown in Table 1. The mean time to reach maximum vacuum during I/A was 1.49 ± 0.45 sec in experienced operators and 2.80 ± 0.98 sec in trainee operators, with a significant difference between two groups (*p* < 0.001). The mean maximum vacuum was 314 ± 70 mmHg in experienced operators and 300 ± 65 mmHg in trainee operators, with no significant difference between two groups (*p* = 0.226). The mean speed of vacuum increase was 225 ± 76 mmHg/sec in experienced operators and 115 ± 35 mmHg/sec in trainee operators and was significantly higher in experienced operators (*p* < 0.001).

Intragroup comparison was also conducted for the group of experienced operators and the group of trainee operators. In the group of experienced operators, time to reach maximum vacuum and speed of vacuum increase were not significantly different between operators, but maximum vacuum was significantly lower in Operator 2 compared to Operators 1 and 3 (both *p* < 0.001). In the group of trainee operators, there were no significant differences in time to reach maximum vacuum, maximum vacuum, and speed of vacuum increase between operators.

For Trainee Operator 1, the time to reach maximum vacuum and speed of increase were 1.54 ± 0.39 sec and 306 ± 29 mmHg/sec, respectively, for the 21st to 40th cataract surgeries (intermediate group) and were 1.50 ± 0.26 sec and 297 ± 32 mmHg/sec for the 41st to 60th cataract surgeries (latter group). The time was significantly reduced and the speed was significantly increased in these subsequent operations compared to the first 20 operations.

### 3.2. Analysis of SMC Graphs and Inappropriate Phacoemulsification Techniques

Analysis of the graphs recorded by SMC revealed inappropriate phacoemulsification techniques. The relationship between SMC parameter data and surgical video is demonstrated in representative examples below.

#### 3.2.1. Inappropriate Nucleus Fragmentation Procedure

**Figure 4:**
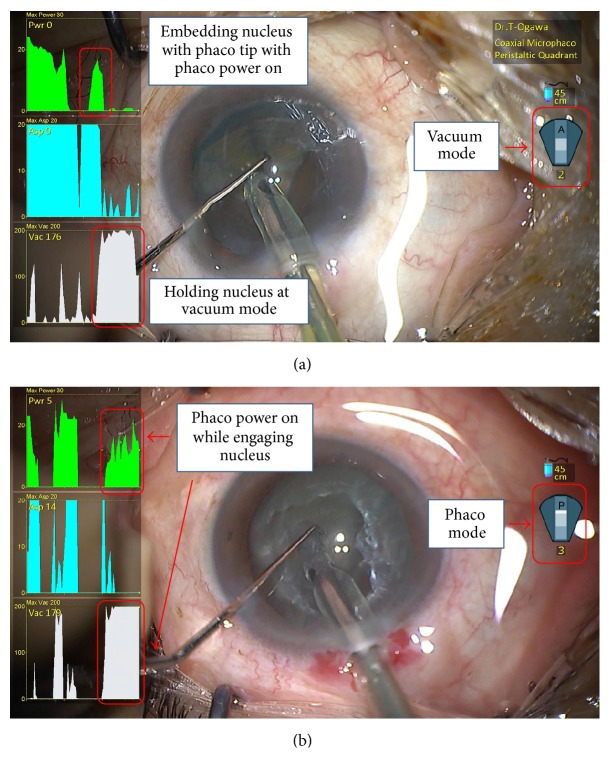
Nucleus fragmentation technique. (a) Correct nucleus fragmentation technique. (b) Case 1, inappropriate nucleus fragmentation technique.

The correct method to fracture the nucleus is first to embed the phaco tip into the nucleus with a short blast of phaco, then stop phaco power, and engage the nucleus under vacuum mode (Figure 4(a)). In a case of inappropriate technique (Case 1, Figure 4(b)), the SMC overlay showed that phaco power remained elevated while the nucleus was being engaged. Hence, SMC revealed that the operator was generating ultrasound while engaging the nucleus.

#### 3.2.2. Inappropriate Cortex Aspiration

**Figure 5:**
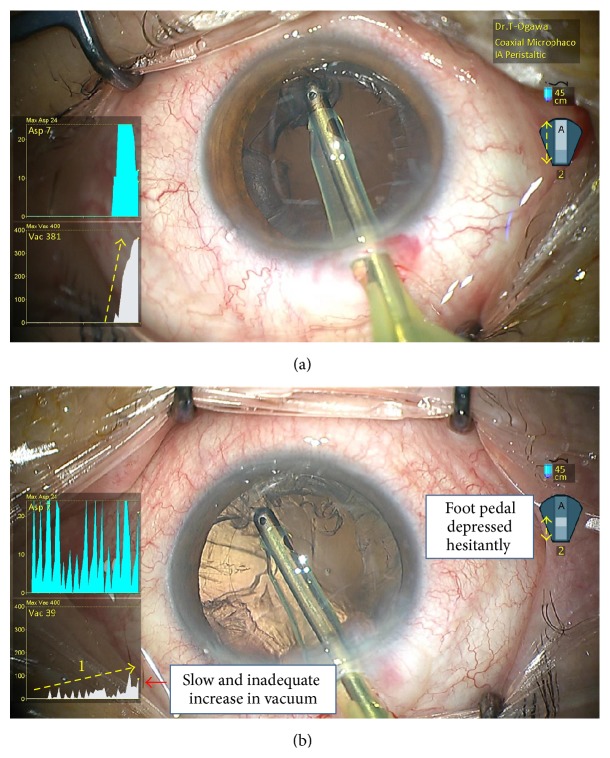
Aspiration of cortex. (a) Appropriate cortex aspiration technique. (b) Case 2, inappropriate cortex aspiration technique.

When cortex aspiration is executed smoothly, vacuum increases steeply reaching a high level within a short time (Figure 5(a)). In Case 2 (Figure 5(b)) in which there was difficulty with cortex aspiration due to insufficient occlusion of the phaco tip and inadequate depression of the foot pedal, the vacuum curve (arrow 1) increased sluggishly reaching a low maximum level.

#### 3.2.3. Inappropriate Trenching

**Figure 6:**
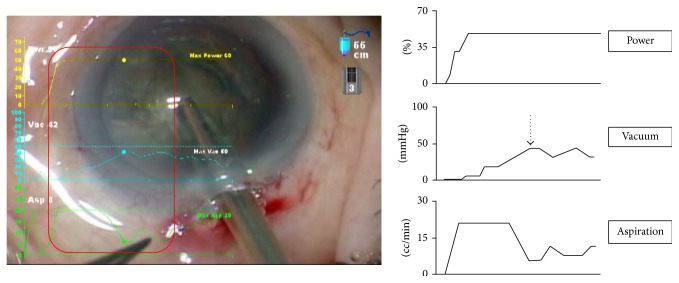
Case 3, trenching performed by trainee operator.

In trenching, nonoccluded aspiration is usually conducted so that the phaco tip does not penetrate the nucleus; hence the vacuum level does not increase. In Case 3 (Figure 6), the vacuum level was elevated (arrow) during trenching, indicating nonoccluded aspiration as not being achieved.

### 3.3. Intraoperative Complication

The causes of intraoperative complications including posterior capsule rupture were examined by analyzing SMC data.

**Figure 7:**
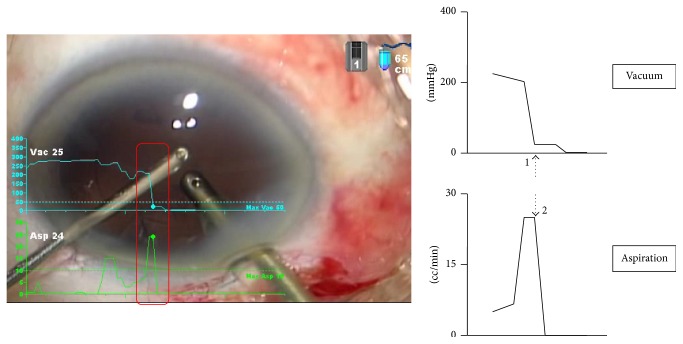
Accidental aspiration of the posterior capsule during aspiration of cortex.

*Posterior Capsule Rupture during Bimanual IA*. In Case 4 (Figure 7), the surgical video depicted only the occurrence of posterior capsule rupture during IA but provided no more information. The SMC overlay, however, showed that while the cortex was aspirated and occlusion at the IA tip was released, the vacuum level dropped (arrow 1) while the aspiration rate increased abruptly (arrow 2). These findings indicated that a surge occurred, which aspirated the posterior capsule accidentally, resulting in posterior capsule rupture.

## 4. Discussion

In SMC and HD-SMC, users can choose any method to display the system data such as phaco power, vacuum level, and aspiration rate. In other words, it is possible to display only those data that are necessary. The format of presentation can be selected either as line graphs (Figure 2) or as bar graphs. The point on the line graph is synchronized with the video image displayed at the same time. Since the data before and after that time point are also displayed, the changes in each parameter over time are clearly depicted. The present study aimed at capturing the techniques and the changes in different parameters during cataract surgery. Therefore we chose to display the data in line graphs that are efficient in demonstrating temporal changes. The *x*- and *y*-axes of these line graphs can also be set at will, and the maximum value for the *y*-axes as well as the speed of progression of the point on the graph can also be selected freely. In other words, when one wishes to inspect the surgical video closely, the graphs can be reduced in size and moved to a location of the screen not overlapping with the video image. When one wishes to examine the changes in the curves in detail, the graph size can be increased and the progression of the graph can be delayed. Depending on what information one desires to obtain from SMC or HD-SMC, the method of display can be selected freely.

Conventional video overlay systems can only display phaco power, vacuum, and aspiration rate of an instant. Therefore the greatest merit of SMC and HD-SMC is the visualization of changes in various parameters over time. When the latest SMC is connected to Signature, the state of foot pedal control can also be visualized, which allows better understanding of the condition of the phacoemulsification device and the operator's manipulations.

Previous reports on cataract surgery education described surgical training using simulations or the necessity of feedback in cataract surgical education [7–16]. There is no report on cataract surgery training using actual intraoperative findings as teaching material. In the present study, we examined the usefulness of SMC and HD-SMC that are capable of displaying detailed and objective surgical parameters in training cataract surgery.

First, we examined whether the surgical recording devices are capable of showing the differences of technical competence between experienced and trainee operators, focusing on the changes in vacuum level during IA. Experienced operators and trainee operators differed significantly in the time taken to reach maximum vacuum and the speed of increase in vacuum level. This difference is also illustrated in Case 2. Because of the lack of experience in cataract surgery, trainee operators are not skillful in operating the foot pedal and hence are unable to control the increase in vacuum by depressing the foot pedal. The SMC recordings showed that trainee operators did not depress the foot pedal sufficiently at the beginning and increased the depressing slowly thereafter. Moreover, surgical videos showed that experienced operators ensured that the IA tip was occluded. Therefore, use of SMC revealed that, in addition to foot pedal control, whether the IA tip is properly occluded also contributes to the difference in speed of vacuum increase.

For Trainee Operator 1, after the first 20 operations, she underwent technical retraining using the SMC aiming to perform IA more effectively. Thereafter, the mean time to reach maximum vacuum, maximum vacuum, and speed of vacuum increase were determined for every 20 consecutive operations. The results showed that as the number of operations increased, the time to reach maximum vacuum was shortened and the speed of increase was improved. These findings imply that, through instructions using the SMC, the operator achieved appropriate pedal control and proper occlusion of the IA tip.

Therefore, the time to reach maximum vacuum and the speed of increase in vacuum may be regarded as indicators for evaluation of the skillfulness of handling the operation device. Educating trainees on the status of pedal control and occlusion of the IA tip based on the values of the above parameters is useful in training surgical techniques.

Using SMC or HD-SMC, inappropriate phacoemulsification techniques were detected; most of the cases were performed by trainee operators.

In Case 1, during nucleus fragmentation, the operator generated ultrasound while trying to engage the nucleus. With the phaco power turned on, the nucleus continued to be fragmented, and it was difficult to stabilize the nucleus with the phaco tip. After the surgery, we reviewed the SMC records with the operator. We found that the operator did not have a good understanding of the principle of nucleus fragmentation, and, during routine surgeries, he was not able to stabilize the nucleus properly and had trouble with nucleus fragmentation. After reviewing the surgical technique using the SMC overlays, he generated ultrasound while embedding the phaco tip into the nucleus; once inside the nucleus, he engaged the nucleus on vacuum mode only. Using this method, he succeeded to conduct nucleus fragmentation reliably.

Case 2 was a case in which cortex aspiration was not smooth. The vacuum curve (arrow 1) increased slowly and reached a low maximum level. The reason was that the operator had not acquired the skills to control the foot pedal and to predict the vacuum response to pedal depression. Worried about accidentally aspirating the capsule, he depressed the foot pedal hesitantly.

Moreover, to increase the vacuum effectively, the IA tip opening has to be occluded properly. The video revealed that the IA tip opening was not occluded sufficiently, which resulted in inadequate increase in vacuum. After viewing the SMC recording, the operator was able to depress the foot pedal stronger than before and ensure that the tip opening was well occluded, achieving more efficient vacuum increase.

In Case 3, a trainee operator performed trenching. Even during trenching, vacuum continued to increase (arrow). Under this condition, nonoccluded aspiration cannot be obtained. In order to perform surgery safety, the operator must ensure complete open aspiration. In addition, decreasing the vacuum setting reduces the risk when occluded aspiration occurs. This case illustrates the usefulness of SMC in reviewing surgical technique and set values for the machine.

By viewing surgical videos, it is possible to have general idea about basic techniques such as manipulation of the hand piece or the hook. However, detailed operations such as the pressure applied to the foot pedal, the intensity of ultrasound generated, and their timing cannot be learnt from conventional surgical videos.

When an operator has problem with performing cataract surgery but does not know the reason, use of the SMC allows the operator to visualize his/her own surgical technique and to compare his/her techniques with the correct techniques. It is easy to comprehend which surgical techniques require improvement. The SMC is thus useful in training surgical techniques and further upgrading of surgical skills.

Moreover, various complications including posterior capsule rupture tend to occur during training. Case 4 illustrates the occurrence of posterior capsule rupture during surgery performed by a trainee operator. The SMC recording clearly showed that posterior capsule rupture was caused by a surge. Using conventional recording devices, one can visualize the occurrence of posterior capsule rupture but cannot understand why it happened. Therefore, there is a possibility that the same complication may occur again in future surgeries. However, reviewing the SMC recording with the operator revealed that the operator lacked knowledge on surge. Taking this opportunity, the operator understood the importance of foot pedal control for surge prevention and was able to put it in practice thereafter. Thus, this case demonstrates that SMC is useful for analyzing the cause of intraoperative complication of posterior capsule rupture and for improving surgical technique.

The present study had a limitation. We used the time taken to reach maximum vacuum and the speed of increase in vacuum as parameters to indicate the technical competence of operators. We hypothesized that skillful control of the foot pedal and timely occlusion of the phaco tip are reflected by a shorter time to reach maximum vacuum and higher speed of vacuum increase. However, further studies are required to investigate the validity of these parameters.

## 5. Conclusion

In the present study, use of SMC or HD-SMC that shows the time courses of phaco power, vacuum, and aspiration rate as well as the state of foot pedal depression superimposed on surgical video allowed more detailed and objective assessments of surgical techniques compared to conventional video overlays. The SMC was able to demonstrate the differences in techniques between experienced and trainee operators, detect inappropriate phacoemulsification techniques by analyzing the graphs, and elucidate the cause of intraoperative complication. Since this recording device allows the review of cataract surgery techniques and identification of the causes of intraoperative complications, it is a valuable tool for educating trainee surgeons on cataract surgery.

## Figures and Tables

**(a):** 

	Time (sec)	Vacuum (mmHg)	Speed (mmHg/sec)
Experienced	1.49 ± 0.45^*∗*a^	314 ± 70	225 ± 76^*∗*d^
Operator 1	1.54 ± 0.38	367 ± 41^*∗*b^	257 ± 90
Operator 2	1.32 ± 0.47	242 ± 50^*∗*bc^	199 ± 62
Operator 3	1.62 ± 0.45	334 ± 47^*∗*c^	222 ± 63
Trainee	2.80 ± 0.98^*∗*a^	300 ± 65	115 ± 35^*∗*d^
Operator 1	2.64 ± 0.68	321 ± 52	126 ± 28
Operator 2	3.42 ± 1.18	311 ± 57	99 ± 30
Operator 3	2.15 ± 1.00	268 ± 74	120 ± 40

^*∗*^ *p* < 0.001, Kruskal-Wallis test. *n* = 20 for each operator. The same superscripted letters indicate that they are compared to each other.

**(b):** 

	Time (sec)	Vacuum (mmHg)	Speed (mmHg/sec)
Trainee Operator 1			
1–20 (operations)	2.64 ± 0.68^*∗*ef^	321 ± 52	126 ± 28^*∗*gh^
21–40 (operations)	1.54 ± 0.39^*∗*e^	306 ± 29	208 ± 44^*∗*g^
41–60 (operations)	1.50 ± 0.26^*∗*f^	297 ± 32	202 ± 36^*∗*h^

^*∗*^ *p* < 0.001, Kruskal-Wallis test. The same superscripted letters indicate that they are compared to each other.
